# Studies on coexistence of *mec* gene, IS256 and novel *sas*X gene among human clinical coagulase-negative staphylococci

**DOI:** 10.1007/s13205-016-0549-9

**Published:** 2016-10-31

**Authors:** K. R. Soumya, Sheela Sugathan, Jyothis Mathew, E. K. Radhakrishnan

**Affiliations:** 1 School of Biosciences, Mahatma Gandhi University, PD Hills (PO), Kottayam, 686 560 Kerala India; 2 MOSC Medical College, Kolencherry, 682 311 Kerala India

**Keywords:** Coagulase-negative staphylococci, Methicillin resistance, *sas*X gene, *mec* gene, IS256

## Abstract

Methicillin-resistant coagulase-negative staphylococci (MRCoNS) are major nosocomial pathogens which cause device-related infections. Presence of various virulence factors along with methicillin resistance favor improved CoNS pathogenicity and their dissemination among population. In the present study, *mec* gene prevalence was analyzed along with SCC *mec* diversity among 55 human clinical CoNS isolates. PCR screening of insertion sequence (IS256) was also conducted to elucidate their association with methicillin resistance among selected CoNS species. In addition to this, PCR screening and sequence analysis of novel colonization-associated gene *sas*X was also carried out. High incidences of *mec* gene, IS256 and their association have been noted among all of the CoNS species tested. Interestingly, eight CoNS isolates were found to harbor *sas*X gene including *S. epidermidis*, *S. hemolyticus* and *S. saprophtyicus* species. Remarkably they were also found to have the coexistence of *mec* gene and IS256 in their genome. Increased SCC *mec* diversity with non-typeable elements was also observed among CoNS isolates. Presence of *sas*X gene in CoNS with *mec* gene and insertion sequence 256 and also the identification of non-typeable SCC *mec* element make the study novel and interesting.

**Electronic supplementary material:**

The online version of this article (doi:10.1007/s13205-016-0549-9) contains supplementary material, which is available to authorized users.

## Introduction

Coagulase-negative staphylococci (CoNS) are major component of the normal flora of human cutaneous ecosystem. In recent years, CoNS have emerged as frequent causative agents of nosocomial illness (Lo et al. 2010). More than 14 species of CoNS are currently recognized as human pathogens and the frequently encountered species are *S. epidermidis, S. hemolyticus, S. hominis,* and *S. saprophyticus* (Kloos et al. 1994). The range of infections caused by CoNS include bacteremia, native and prosthetic valve endocarditis, osteomyelitis, pyoarthritis, peritonitis and implanted medical device-related infections (Huebner and Goldmann 1999). The remarkable biofilm formation and ability to acquire and disseminate multidrug resistance have been considered to contribute significantly to the emergence and propagation of CoNS in nosocomial and community settings (Longauerova 2006; Duran et al. 2012). The biofilm formation in CoNS can either be of *ica*ADBC-mediated polysaccharide type or of proteinaceous type (von Eiff et al. 2002) and this reduces bacterial susceptibility to drugs.

As CoNS are part of commensal flora, the presence of drug resistance in them is highly dangerous. The mechanism of resistance to penicillin is mainly dependent on the expression of the *mec*A gene, which encodes PBP2a transpeptidase with low affinity towards most of the semisynthetic penicillins (Duran et al. 2012). Remarkably *mec*A gene is present as a part of staphylococcal cassette chromosome *mec* element (SCC *mec*). SCC *mec* composed of *mec* gene complex, *ccr* gene complex and three joining (J) regions. Based on the classes of the *mec* gene complex and the *ccr* gene type there are 11 different SCC *mec* types for *S. aureus* which itself highlights its importance. But SCC *mec* elements are likely to be more diverse in MRCoNS due to continuous generation of new composite cassettes that do not fit within the classification proposed for MRSA (Lo et al. 2010; Lebeaux et al. 2011; Zong et al. 2011). Another genetic element, the insertion sequence IS256 which is mainly involved in phase variation of biofilm forming CoNS has been identified to have coexistence with *mec* gene (Kozitskaya et al. 2005). Autonomous movement and multiple independent insertions of this element into the staphylococcal chromosome together with point mutations, homologous recombination, and horizontal gene transfer act as driving forces for the generation of novel genetic and phenotypic variants. Role of IS256 in association with methicillin heteroresistance in *S. aureus* and the modulation of methicillin resistance in *S. sciuri* has also been suggested (Maki and Murakami 1997; Couto et al. 2003). Hence studies on coexistence of *mec* gene and IS256 among CoNS from clinical samples are highly interesting and significant.

Genetic analysis on association of *mec*A and IS256 with other newly identified virulence factors may provide deeper insight into emerging molecular programming which is being conducted in CoNS. The novel *sas*X gene with a signal peptide and LPXTG surface anchoring motif identified from *S. aureus* with ΦSPβ-like prophage is very significant in this context. No other orthologues for this gene have been reported except in *S. epidermidis* RP62A ΦSPβ-like prophage which has 95.1% amino acid identity to the *sesI* gene associated with the clinical strains with invasive properties. SasX provide advantages to the organism due to its role in increased nasal colonization onto host, immune evasion, virulence, bacterial aggregation, biofilm formation and even attachment to the abiotic surfaces (Holden et al. 2010; Li et al. 2012; Otto 2012). In our previous study, we could identify the presence of *sas*X gene in *S. epidermidis* (Soumya et al. 2016). Hence in this study, prevalence of *sas*X gene was analyzed among methicillin-resistant clinical isolates of coagulase-negative staphylococci to investigate its relation to *mec*A gene and IS256.

PCR based molecular analysis for the coexistence of *mec* gene, IS256 and *sas*X gene and also SCC *mec* typing was carried out for CoNS from human clinical samples. The result showed remarkable coexistence and relation between selected genes and also presence of untypeable SCC *mec* elements in CoNS which provide precautions about the likely role of CoNS to further disseminate these to other microorganisms of human microbiome.

## Materials and methods

### Strains used in this study

A total of 100 coagulase-negative staphylococci from different clinical samples including exudates, urine, blood, catheter tips, and sputum were collected from a tertiary care hospital in Ernakulam, Kerala, India. The clinical isolates from nutrient agar slopes were quadrantly streaked on to tryptic soy agar plates and incubated for 24–48 h. The colonies observed as coagulase-negative staphylococci on the basis of colony morphology were selected and these isolates were identified using Gram staining, coagulase test and biochemical tests (unpublished data). Methicillin resistance were initially screened using oxacillin and cefoxitin disc diffusion assays, from these 55 MRCoNS strains were obtained which were further selected for this study. Genomic DNA was extracted using the Bacterial genomic DNA isolation Mini Spin kit (Chromous Biotech, Bangalore) following the manufacturer’s instructions. Molecular identification of CoNS species was conducted using Multiplex PCR (MPCR). Here, identification of 55 CoNS were performed by amplifying the *nuc* and adjacent genes using species specific primers (Hirotaki et al. 2011). 4 CoNS isolates which were not amplified in MPCR were further subjected to 16SrDNA sequencing (Chun and Goodfellow 1995).

### PCR screening for *mec*A gene, *IS*256 and *sas*X gene

Separate PCRs were conducted for the detection of *mec*A gene, IS256 and *sas*X. For the detection of *mec*A, gene the primers used were *mec*A-F, (5′-GAAATGACTGAACGTCCGAT-3′) and *mec*A-R, (5′-GCGATCAATGTTACCGTAGT-3′) to amplify a 154-bp gene fragment (Rohde et al. 2004). The presence of IS256 was screened by using IS256-F (5′-TGAAAAGCGAAGAGATTCAAAGC-3′) and IS256-R (5′ATGTAGGTCCATAAGAACGGC-3′) primers (Ziebuhr et al. 1997).

Amplification of a 522-bp fragment of *sas*X gene was carried out using the primers *sas*X-F (5′-AGAATTAGAAGTACGTCTAAATGC3′) and *sas*X-R (5′GCTGATTATGTAAATGACTCAAATG-3′) (Li et al. 2012). In all the PCR reactions, the 50 µL PCR mix contained, 5 µL of 10× PCR buffer, 4 µL of dNTPs (200 µM), 0.5 units of *Taq* DNA polymerase, 4 µL (5 pmol) of specific primers and 6 µL of DNA template. The volume was made up to 50 µL with sterile MilliQ water. PCR was conducted on Mycycler™ (Bio-Rad, USA) with initial denaturation for 5 min at 94 °C, followed by 35 cycles of denaturation at 94 °C for 30 s, annealing at primer specific temperature for 30 s and elongation at 72 °C for 1 min, with a final extension at 72 °C for 4 min. The amplified products were analyzed on 1.5% of agarose gel and were visualized on UV transilluminator. To confirm the identity of the PCR amplicons and to analyze sequence variations among staphylococcal species, all of the *sas*X PCR products were further gel purified and sequenced using Big Dye Terminator Sequence Reaction Ready Mix (Applied Biosystem). The sequence data obtained were further subjected to BLAST analysis.

### Determination of SCC *mec* types

For this, a total of 20 CoNS (four *S. hominis*, three *S. hemolyticus*, one *S. sciuri*, one *S. cohnii*, two *S. saprophyticus* and nine *S. epidermidis)* harboring *mec* gene were subjected to SCC *mec* typing using Multiplex SCC *mec* typing MRSA Detection kit (HiMedia, Mumbai). As per manufacture’s protocol the PCR was conducted in Sure cycler 8800 (Agilent) and the obtained amplicons were visualized on 2% agarose gel.

## Results

From a total of 100 CoNS isolates processed, 55 isolates which showed methicillin resistance on antibiotic sensitivity assay were selected for the study. Among 55 MRCoNS, 51 isolates showed amplification specific for different CoNS species. 31 isolates showed amplification corresponding to *S. epidermidis* (251 bp) followed by 13 *S. hemolyticus* isolates (434 bp). Amplification of *nuc* gene resulted in 843-bp product for five isolates specific to *S. saprophyticus*, whereas other four isolates showed 177-bp product specific for the species *S. hominis*. Rest of the MPCR unidentified clinical isolates were subjected to 16S rDNA partial sequencing and the results were BLAST analyzed. Two of the isolates were identified with 100% identity to *S. hemolyticus* and other two isolates showed maximum identity with *S. sciuri* and with *S. cohnii*. Thus *S. epidermidis* (*n* = 31) showed 56% distribution followed by *S. hemolyticus* (*n* = 13, 24%), *S. saprophyticus* (*n* = 5, 9%), *S. hominis* (*n* = 4, 7%), *S. sciuri* (*n* = 1, 2%), *S. cohnii* (*n* = 1, 2%) (Supplementary Figure 1).

### PCR screening for *mec*A gene, *IS*256 and *sas*X gene among CoNS clinical isolates

**Fig. 1:**
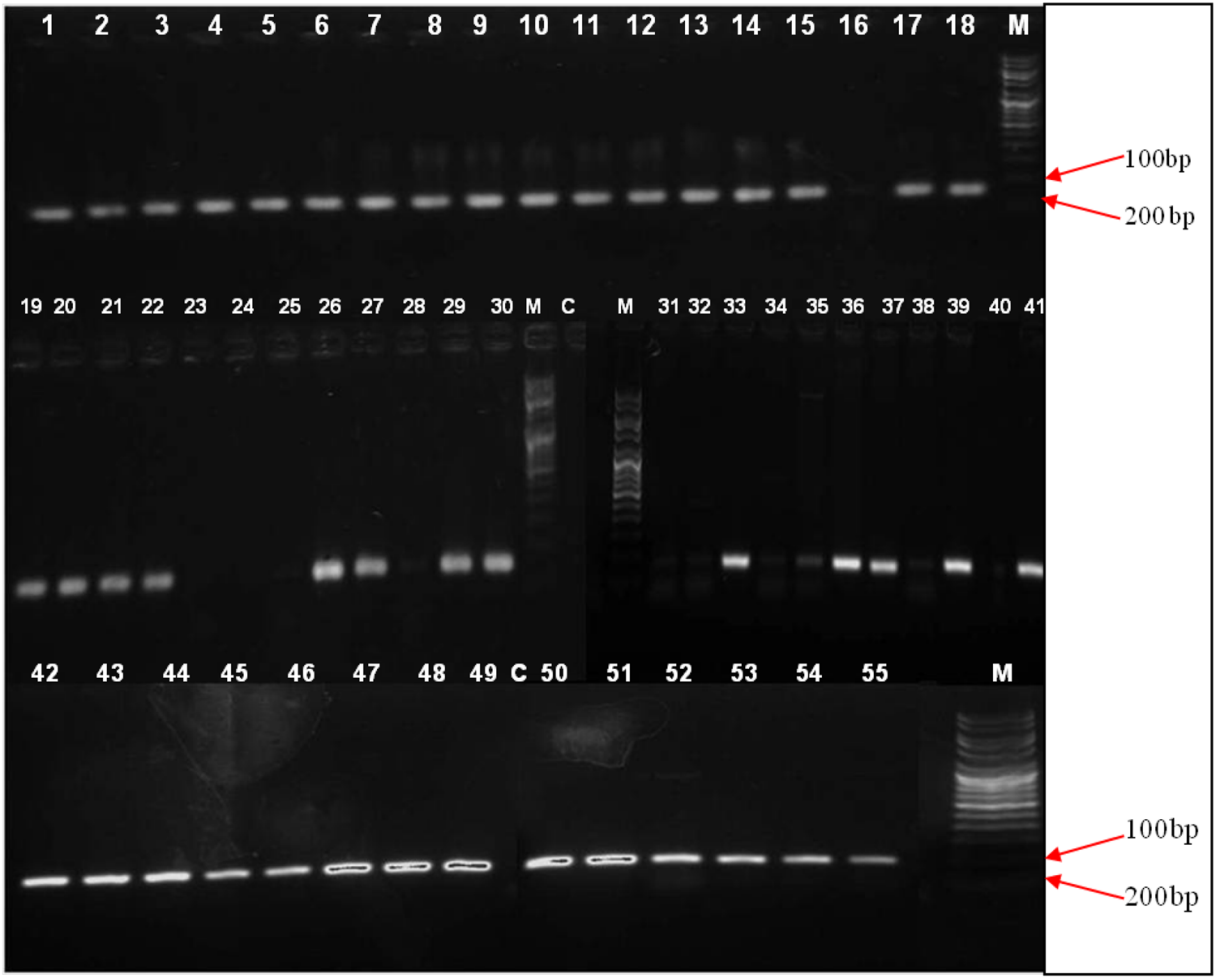
PCR amplification of *mec*A gene. Lanes 1–55 - CoNS isolates, M - DNA molecular weight marker, C - negative control. *mec*A gene amplification corresponds to 154 bp product

**Fig. 2:**
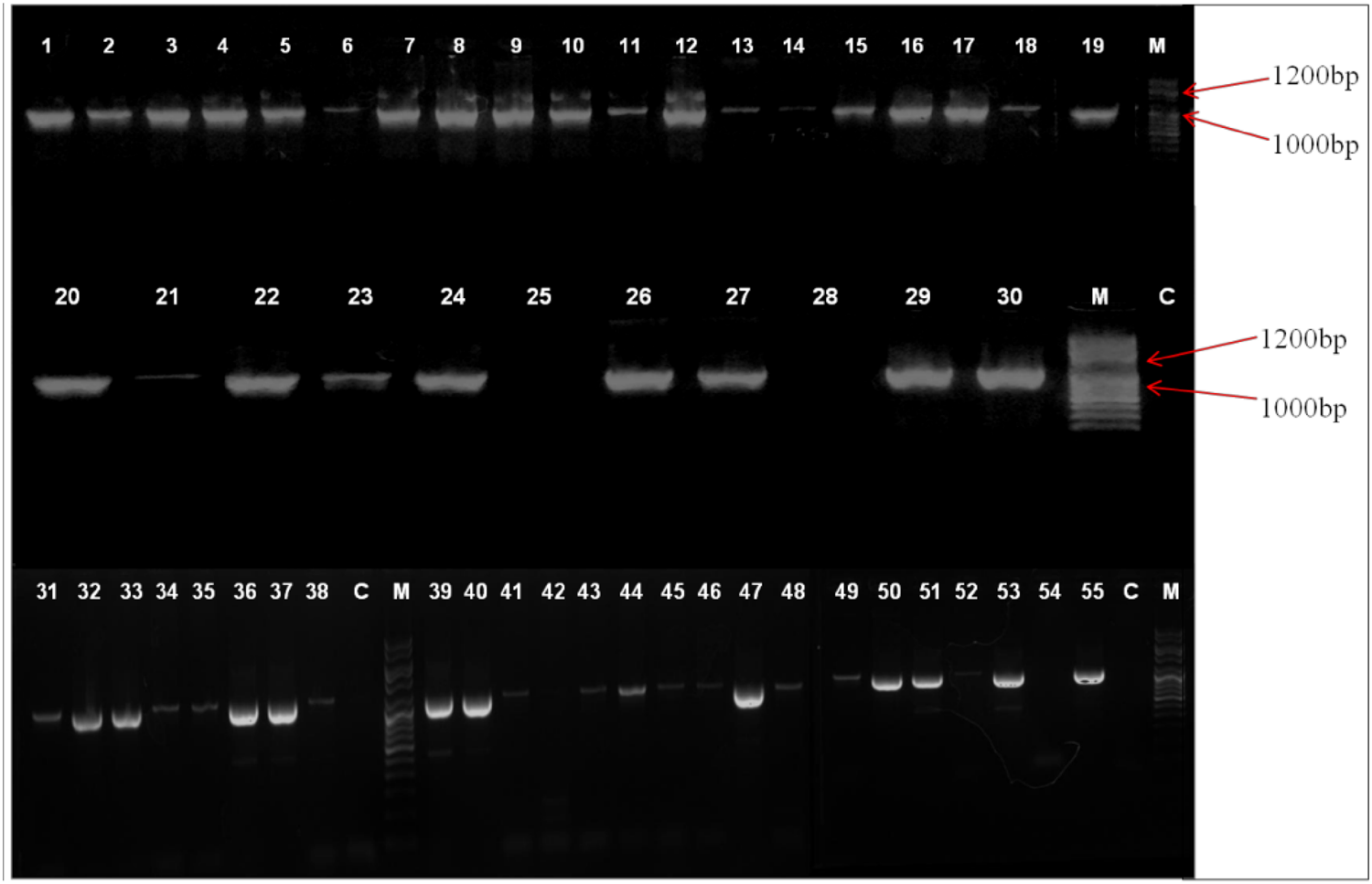
PCR identification of IS256 among 55 CoNS species. Lanes 1–55 - CoNS isolates, M - DNA molecular weight marker, C - negative control. IS256 amplification is indicated by 1102 bp product

PCR results confirmed 53 CoNS isolates (96%) to have *mec* gene and IS256 individually, whereas 52 (95%) isolates were found to have the presence of both the genes. Among tested *S. epidermidis*, 97% amplified *mec* gene whereas 94% were found to be IS256 positive. All of the 13 *S. hemolyticus* strains harbored IS256 but *mec* gene absence was observed for one of the *S. hemolyticus* isolate. Rest of the CoNS isolates tested in this study (*S. saprophyticus*, *S. hominis*, *S. sciuri* and *S. cohnii*) were found to have the coexistence of both the *mec* gene and IS256 in their genome. Distribution of these genes among CoNS is described in Table 1, Figs. 1 and 2.

**Table 1:** Distribution of *mec* gene, IS256 and *sas*X gene among CoNS species

CoNS species	*mec* gene	IS256	*sas*X gene
*S. epidermidis*	30	29	4
*S. hemolyticus*	12	13	3
*S. saprophyticus*	5	5	1
*S. hominis*	4	4	–
*S. sciuri*	1	1	–
*S. cohnii*	1	1	–

**Fig. 3:**
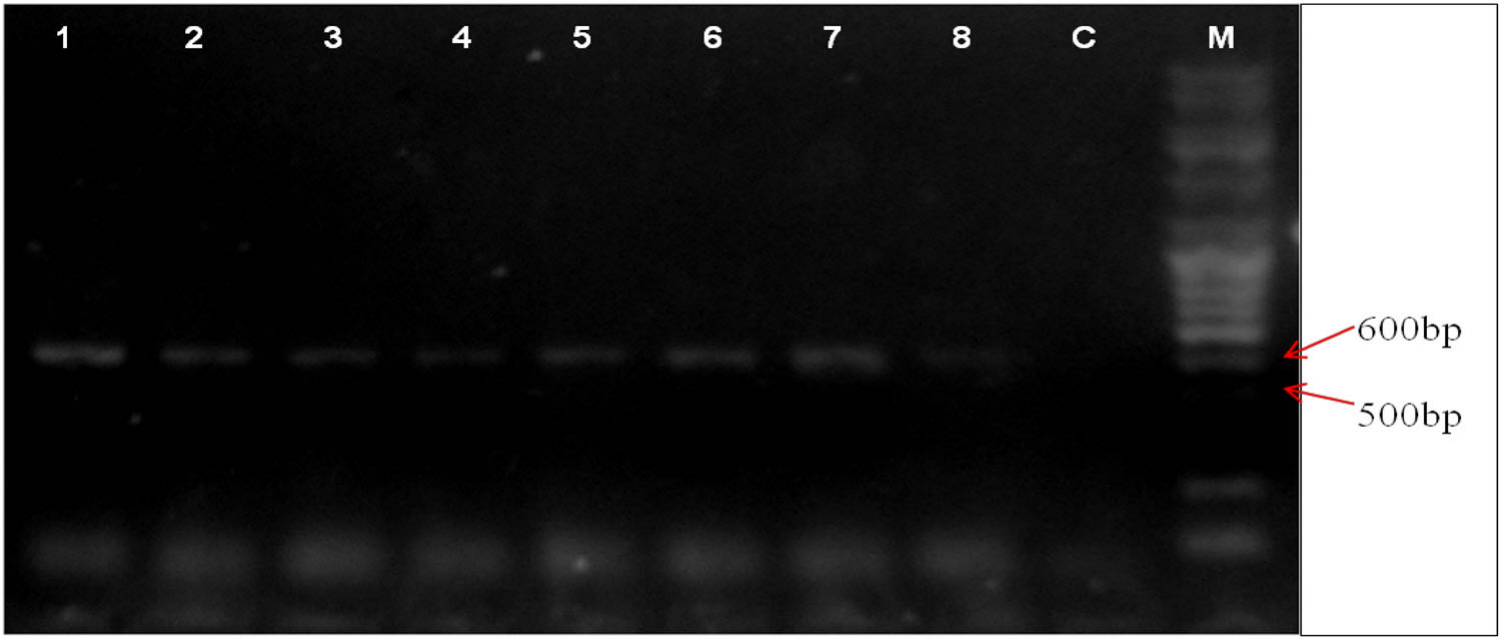
PCR identification of *sas*X gene. Lanes 1-8 represent *sas*X gene amplification at 522 bp for CoNS isolates, M - DNA molecular weight marker, C - negative control

Among 55 samples analyzed, eight isolates showed presence of product of 522 bp size corresponding to the size of *sas*X gene and hence these were further sequenced, analyzed by BLAST and compared with the *sas*X gene of *S. aureus* TW20. Interestingly, these eight isolates also harbored *mec* gene and IS256 within their genome. Specifically, four *S. epidermidis* isolates, three *S. hemolyticus* and one *S. saprophyticus* were found to possess this gene. Translated *sas*X gene sequence of three *S. hemolyticus*, two *S. epidermidis* and one *S. saprophyticus* showed 100% identity to the LPXTG sequence of *S. aureus* TW20, whereas other two *S. epidermidis* isolates showed only 98 and 95% identities to the reference sequence (Fig. 3).

### SCC *mec* typing using MPCR assay Kit

**Fig. 4:**
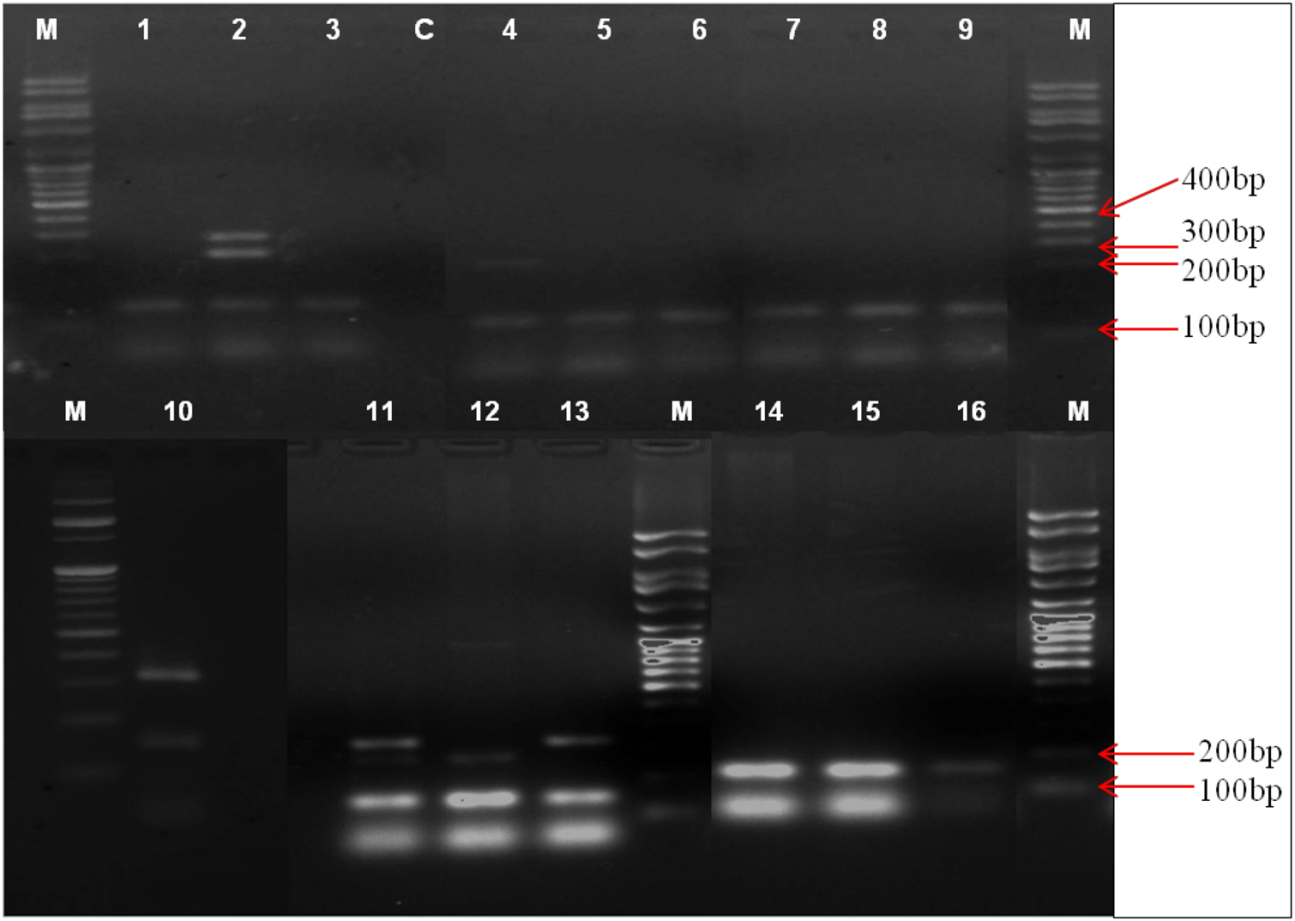
PCR identification of *SCC mec* types. M - DNA molecular weight marker, C - negative control. Lanes 1–16 - CoNS isolates with an amplification at 147 bp corresponding to *mec* gene. Lanes 4 and 12 SCC *mec* type III with amplification at 280 bp. Lanes 2 and 10 multiple amplification at 280 bp 325 bp correspond to SCC *mec* type III and V, respectively. Lanes 11 and 13 amplification at 325 bp indicating the presence of SCC *mec* type V

SCC *mec* typing has been carried out for selected isolates from each species using the multiplex kit. Of 20 isolates tested, 16 isolates showed amplification for the *mec* gene. Only six isolates showed the presence of SCC *mec* specific amplification pattern. Three *S. epidermidis* belonged to different SCC *mec* types. Two of the *S. epidermidis* isolates had multiple amplifications at 280bp and 325 bp which indicated the occurrence of both the SCC *mec* type III and V, respectively, within these isolates, whereas another isolate had an amplification corresponding to SCC *mec* type III only. One of the *S. hemolyticus* isolates showed the amplification corresponding to the SCC *mec* type V. In the case of *S. hominis,* one isolate showed amplification corresponding to SCC *mec* type III and other isolate to  type V. (Fig. 4).

## Discussion

Even though coagulase-negative staphylococci were considered as harmless skin commensals, their frequent isolation from various clinical samples has changed its status to nosocomial pathogens. As MRCoNS are widespread in all parts of the world, it represent a serious burden of infection in both nosocomial and community settings. Various factors involved in CoNS pathogenesis are now being explored; major among them is methicillin resistance and associated virulence determinants (Ibrahem et al. 2009).

In this study, 55 MRCoNS were selected from clinical samples which belonged to six different CoNS species. Among these 30 *S. epidermidis* were found to carry *mec* gene. Various reports on methicillin carriage in *S. epidermidis* showed it to have a distribution rate of 69–84% (Lebeaux et al. 2011) and is in accordance with the results of current study. In addition, all of the *S. hominis*, *S. saprophyticus*, *S. cohnii* and *S. sciuri* isolates were also found to have the presence of *mec* gene. Human clinical isolates of these species were already demonstrated to have *mec* gene with diverge SCC *mec* types (Mombach Pinheiro Machado et al. 2007). Out of the 13 methicillin-resistant *S. hemolyticus* identified in the present study, 12 were identified to have the association of *mec* gene which confirms their major role in promoting antibiotic resistance phenotypes (Diekema et al. 1999).

Among 20 isolates tested using SCC *mec* multiplex identification kit, 16 showed *mec* gene amplification and 6 of the isolates showed the presence of SCC *mec* elements. Apart from the common classical *mec*A gene, different variants *mec*A1, *mec*A2, *mec*C and *mec*C2 have also been reported from CoNS species of both human and animal origin and the polymorphism can expected to have determining effect on its positive result in the PCR screening (Becker et al. 2014). SCC *mec* diversity in staphylococci is very common and till now almost 11 SCC types and various subtypes has been reported. In this study two *S. epidermidis* isolates showed amplification for multiple SCC types-III and V, also two *S. hominis* isolates individually  showed the presence of  SCC *mec* type III and V. Another single isolate of *S. epidermidis*  showed the presence of SCC *mec* type III and  one *S. hemolyticus* isolate was found to be PCR positive for SCC *mec* type V. These results were in accordance with the previous reports suggesting the high prevalence of SCC *mec* type III, IV and V among CoNS. Non-typeable *mec* elements are frequent in CoNS and this may be the reason for the absence of SCC *mec* types among the other CoNS isolates tested in this study (Zong and Lü 2010).

High degree of coexistence of IS256 with *mec* gene was also identified for all the CoNS species used in this study. More specifically, 29 *S. epidermidis* isolates, 12 *S. hemolyticus* isolates and all other CoNS isolates were found to carry both IS256 and *mec* genes in their genome. Overall 95% of the CoNS isolates showed the association of these genes and the result was in accordance with previous report. IS256 has also been suggested as a good marker gene to differentiate between invasive and noninvasive nature of CoNS isolates due to its frequent association with multidrug-resistant isolates (Mertens and Ghebremedhin 2013). Mechanistically, IS256 transpositions have the potential to create new hybrid promoter with enhanced transcription with concomitant elevation of the methicillin resistance (Maki and Murakami 1997).

Further analysis of coexistence of *sas*X gene along with IS256 and *mec* gene showed the presence of novel *sas*X gene among *S. epidermidis*, *S. hemolyticus* and *S. saprophyticus* species. Its existence in *S. epidermidis* was already reported in our previous work (Soumya et al. 2016). However, the presence of this gene in *S. hemolyticus* and *S. saprophyticus* species is a novel report and this was confirmed by sequence analysis which showed 100% percentage identity to the LPXTG motif of *S. aureus* TW20. Even though two of the *S. epidermidis* isolates were 100% similar to the reference gene, other two isolates showed only 98 and 95% similarity. Interestingly the eight *sas*X-positive isolates identified in this study were also positive for *mec* gene and IS256 which indicate the possibility to evolve CoNS as potential virulent pathogens. The presence of *sas*X gene can enhance the colonization property of CoNS and once this occurs *mec* gene and IS256 further promote rapid propagation of the organism resulting in serious infections. Also the presence of *sas*X gene among different CoNS species identified in the study indicates the possible horizontal transfer of the property which was earlier suspected to occur from *S. aureus* (Otto 2012).

The high degree of coexistence of methicillin resistance and IS256 sequence among different species of CoNS indicates their role in infectious disease. Also the colonization promoting *sas*X gene can favor these characteristics which can lead even to their asymptomatic carriage among community settings. These findings highlight the importance of the present work which provide molecular insight into the prevalence of genes which facilitate survival and pathogenesis of CoNS species in the human host which can ultimately contribute to the virulence potential of these dangerous pathogens.

## Electronic supplementary material

Below is the link to the electronic supplementary material.

Supplementary material 1 (DOCX 401 kb)
