# Supplementary material for: Studies on coexistence of mec gene, IS256 and novel sasX gene among human clinical coagulase-negative staphylococci
Source: 3 Biotech. 2016 Oct 31;6(2):233. doi: 10.1007/s13205-016-0549-9 (PMC5088179; doi:10.1007/s13205-016-0549-9)
Supplement: Supplementary file 1 — Supplementary material 1 (DOCX 401 kb) [file 13205_2016_549_MOESM1_ESM.docx]

**Studies on co-existence of *mec* gene, IS256 and novel *sas*X gene among human clinical coagulase negative staphylococci**

KR Soumya^1^, Sheela Sugathan^2^, Jyothis Mathew^1^ and EK Radhakrishnan^1*^

^1^School of Biosciences, Mahatma Gandhi University, PD Hills (PO), Kottayam, Kerala, India – 686 560

^2^MOSC Medical College, Kolencherry, Kerala, India- 682 311

^*^ Corresponding author: [radhakrishnanek@mgu.ac.in](mailto:radhakrishnanek@mgu.ac.in)

Tel/fax: +91 9847901149

**Supplementary figure**

**
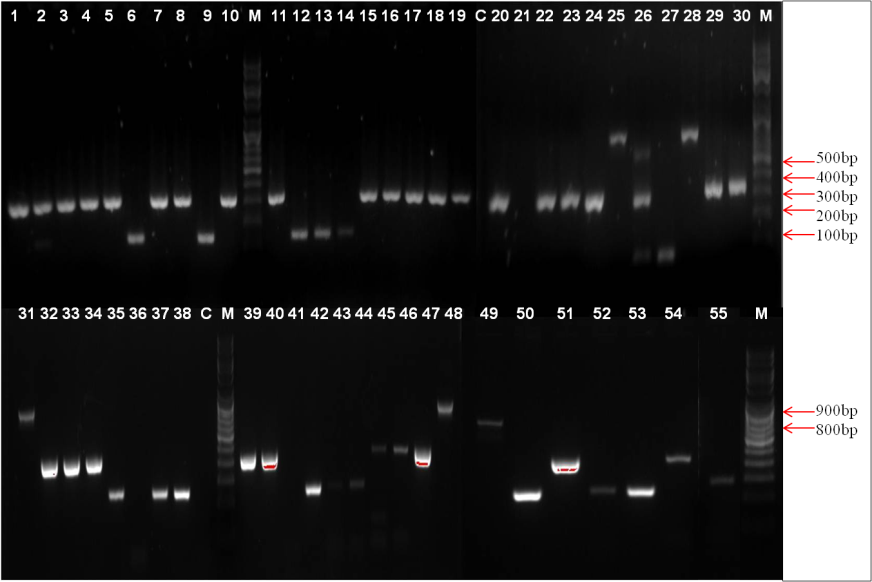
**

**Supplementary Figure 1: Multiplex PCR species specific identification of 55 CoNS species.** Lane 1-55: CoNS isolates, M: DNA molecular weight marker (100bp), C: negative control. *S. epidermidis* showed amplification at 251bp, *S. hemolyticus* at 434bp, *S. hominis* at 177bp and *S. saprophyticus* at 843bp

**31 32 33 34 35 36 37 38 C M 39 40 41 42 43 44 45 46 47 48 49 50 51 52 53 54 55 M**

**1 2 3 4 5 6 7 8 9 10 M 11 12 13 14 15 16 17 18 19 C 20 21 22 23 24 25 26 27 28 29 30 M**
